# Supplementary material for: Different B cell subpopulations show distinct patterns in their IgH repertoire metrics
Source: eLife. 2021 Oct 18;10:e73111. doi: 10.7554/eLife.73111 (PMC8560093; doi:10.7554/eLife.73111)
Supplement: Supplementary file 1. [file elife-73111-supp1.docx]

| **Participant ID** | **Cells** | **Age** | **Sex** | **B cell number** | **Purity** |
| --- | --- | --- | --- | --- | --- |
| Co_C.081.1_BC | MZB | 50 | M | 250000 | 93.6 |
| Co_C.081.1_BC | Naive | 50 | M | 250000 | 96.7 |
| Co_C.080.1_BC | MZB | NA | M | 250000 | NA |
| Co_C.080.1_BC | Naive | NA | M | 250000 | NA |
| Co_C.082.1_BC | Naive | 40 | M | 250000 | 97.6 |
| Co_C.083.1_BC | MZB | 18 | M | 250000 | 82.9 |
| Co_C.083.1_BC | Naive | 18 | M | 250000 | 98.2 |
| Co_C.084.1_BC | MZB | 36 | F | 250000 | 86.4 |
| Co_C.084.1_BC | Naive | 36 | F | 250000 | 96.7 |
| Co_C.081.1_BC | Swt | 50 | M | 250000 | 98.8 |
| Co_C.080.1_BC | Swt | NA |  | 250000 | NA |
| Co_C.080.1_BC | PC | NA |  | 55000 | NA |
| Co_C.081.1_BC | PC | 50 | M | 1.00E+05 | 45.8 |
| Co_C.082.1_BC | MZB | 40 | M | 250000 | 81.5 |
| Co_C.082.1_BC | Swt | 40 | M | 250000 | 98.7 |
| Co_C.082.1_BC | PC | 40 | M | 19000 | 67.8 |
| Co_C.083.1_BC | Swt | 18 | M | 250000 | 99.2 |
| Co_C.083.1_BC | PC | 18 | M | 21000 | 32.8 |
| Co_C.081.1_BC | CD19 | 50 | M | 5.00E+05 | NA |
| Co_C.084.1_BC | Swt | 36 | F | 250000 | 98.2 |
| Co_C.084.1_BC | PC | 36 | F | 15000 | 30.3 |
| Co_C.084.1_BC | CD19 | 36 | F | 5.00E+05 | NA |
| Co_C.085.1_BC | CD19 | 41 | M | 5.00E+05 | NA |
| Co_C.085.1_BC | PC | 41 | M | 21000 | 32.2 |
| Co_C.085.1_BC | Swt | 41 | M | 250000 | 97.5 |
| Co_C.085.1_BC | Naive | 41 | M | 250000 | 98.9 |
| Co_C.085.1_BC | MZB | 41 | M | 250000 | 92.1 |
| Co_BC7_BC | Naive | 49 | F | 250000 | 93.6 |
| Co_BC8_BC | MZB | 59 | F | 250000 | 90.5 |
| Co_BC8_BC | Naive | 59 | F | 250000 | 95.2 |
| Co_BC9_BC | MZB | 44 | F | 250000 | 91.8 |
| Co_BC9_BC | Naive | 44 | F | 250000 | 99.2 |
| Co_BC10_BC | MZB | 51 | F | 250000 | 94.2 |
| Co_BC10_BC | Naive | 51 | F | 250000 | 96.2 |
| Co_BC7_BC | CD19 | 49 | F | 5.00E+05 | NA |
| Co_BC7_BC | Swt | 49 | F | 250000 | 95.6 |
| Co_BC7_BC | PC | 49 | F | 14000 | 37 |
| Co_BC8_BC | CD19 | 59 | F | 5.00E+05 | NA |
| Co_BC8_BC | Swt | 59 | F | 250000 | 97.3 |
| Co_BC8_BC | PC | 59 | F | 24000 | 68.2 |
| Co_BC9_BC | CD19 | 44 | F | 5.00E+05 | NA |
| Co_BC9_BC | Swt | 44 | F | 250000 | 94.5 |
| Co_BC9_BC | PC | 44 | F | 22000 | 82.8 |
| Co_BC10_BC | CD19 | 51 | F | 5.00E+05 | NA |
| Co_BC10_BC | Swt | 51 | F | 250000 | 99.1 |
| Co_BC10_BC | PC | 51 | F | 19000 | 60 |
| Co_C.082.1_BC | CD19 | 40 | M | 5.00E+05 | NA |
| Co_BC7_BC | MZB | 49 | F | 250000 | 86.7 |
| Co_C.083.1_BC | CD19 | 18 | M | 5.00E+05 | NA |
